# Clinical significance of primary tumor score determined by tumor depth and size in patients with resectable gastric cancer

**DOI:** 10.18632/oncotarget.23953

**Published:** 2018-01-04

**Authors:** Naoto Haraguchi, Takaaki Arigami, Yoshikazu Uenosono, Shigehiro Yanagita, Yasuto Uchikado, Shinichiro Mori, Hiroshi Kurahara, Yuko Kijima, Akihiro Nakajo, Kosei Maemura, Sumiya Ishigami, Shoji Natsugoe

**Affiliations:** ^1^ Department of Digestive Surgery, Breast and Thyroid Surgery, Kagoshima University Graduate School of Medical and Dental Sciences, Kagoshima, Japan; ^2^ Onco-biological Surgery, Kagoshima University Graduate School of Medical and Dental Sciences, Kagoshima, Japan

**Keywords:** primary tumor score, tumor size, depth of tumor invasion, prognostic factor, gastric cancer

## Abstract

Although postoperative management of gastric cancer is determined by pathological stage based on the tumor-node-metastasis classification, predicting disease recurrence and prognosis in patients undergoing gastrectomy is clinically difficult. We investigated the depth of tumor invasion and tumor size in resected specimens from patients with gastric cancer and assessed the clinical utility of primary tumor score (PTS) calculated by tumor depth and size as a prognostic marker. We classified 247 patients with gastric cancer into three groups based on cut-off values for deeper tumor invasion (pT2–T4) and larger tumor size (≥ 45 mm) as a PTS of 2 (both abnormalities), 1 (one abnormality), or 0 (neither abnormality). PTS correlated significantly with lymph node metastasis, lymphovascular invasion, and stage (*P* < 0.0001 each). Survival differences among groups based on PTS were significant (*P* < 0.0001). Multivariate analysis identified PTS alone as an independent prognostic factor (*P =* 0.0363). PTS derived from primary tumor information alone is a potentially useful marker for predicting tumor progression and prognosis in postoperative patients with gastric cancer.

## INTRODUCTION

Gastric cancer is a common gastrointestinal malignancy and the third leading cause of cancer death globally [1]. Although the prognosis of patients with resectable gastric cancer has improved due to the remarkable progress in surgical techniques and chemotherapies, some patients die of recurrent disease after surgery. The need for administration of adjuvant chemotherapy is currently judged based on pathological stage in patients with resectable gastric cancer [2]. According to the criteria of the Japanese Gastric Cancer Treatment Guidelines 2014 (ver. 4), induction of adjuvant chemotherapy is recommended for patients with stage II or III pathology [2]. This therapeutic strategy indicates a high incidence of disease recurrence in postoperative patients with stage II-III. On the other hand, carcinoembryonic antigen (CEA) and carbohydrate antigen 19-9 (CA19-9) are representative blood markers for the clinical management of patients with gastric cancer. However, the sensitivity and specificity of these conventional tumor markers are clinically insufficient for predicting disease recurrence and prognosis [3, 4]. Consequently, these key issues suggest few prognostic indicators are available for accurately predicting survival outcomes in patients with gastric cancer.

In postoperative management, patients are pathologically categorized and staged based on the criteria of the tumor-node-metastasis (TNM) classification of gastric carcinoma established by the Union for International Cancer Control (UICC) [5]. Pathological TNM stage involving tumor progression and prognosis is comprehensively decided based on the depth of tumor invasion and the presence of lymph node metastasis and distant metastasis [5]. Accordingly, we need to grasp three different clinicopathological statuses in order to determine pathological stage based on this classification system.

Several investigators have demonstrated the clinical impact of tumor size as a simple prognostic indicator in patients with resectable gastric cancer [6–15]. Furthermore, according to a large-scale retrospective analysis of 2405 patients with gastric cancer, tumor size could improve the accuracy of TNM staging in predicting survival [15]. However, few reports have revealed that tumor depth and size have been simultaneously assessed as a predictive marker for tumor progression and prognosis in patients with resectable gastric cancer [16]. If a primary tumor score (PTS) based on both tumor depth and size correlated with TNM stage, we may be able to predict survival outcomes using primary tumor status alone, even without knowing disease status in terms of lymph node metastasis or distant metastasis.

The present study examined tumor depth and size in patients with resectable gastric cancer, and evaluated the relationship between these primary tumor factors and clinicopathological factors. We also assessed the potential utility of PTS based on tumor depth and size as a prognostic indicator for gastric cancer.

## RESULTS

### Tumor depth and size as predictors of tumor progression

We initially examined the relationship between clinicopathological factors and depth of tumor invasion or tumor size to assess their clinical impact during tumor progression in 247 patients with resectable gastric cancer.

Pathological examination demonstrated pT1 in 138 patients, pT2 in 17 patients, pT3 in 55 patients, and pT4 tumors in 37 patients. Table 1 shows the relationship between status of tumor depth and clinicopathological features. Tumor depth correlated significantly with lymph node metastasis, lymphatic and venous invasions, and stage (*P* < 0.0001 each).

**Table 1:** Relationship between depth of tumor invasion and clinicopathological factors

Factor	Depth of tumor invasion				*P*-value
	pT1 (*n =* 138)	pT2 (*n =* 17)	pT3 (*n =* 55)	pT4 (*n =* 37)	
Sex					
Male	90 (65.2)	14 (82.3)	37 (67.3)	23 (62.2)	0.5045
Female	48 (34.8)	3 (17.7)	18 (32.7)	14 (37.8)	
Age (y)					
≤ 70	72 (52.2)	11 (64.7)	29 (52.7)	25 (67.6)	0.3139
>70	66 (47.8)	6 (35.3)	26 (47.3)	12 (32.4)	
Lymph node metastasis					
Negative	114 (82.6)	8 (47.1)	14 (25.4)	7 (18.9)	< 0.0001
Positive	24 (17.4)	9 (52.9)	41 (74.6)	30 (81.1)	
Stage					
I	130 (94.2)	9 (52.9)	0 (0.0)	0 (0.0)	< 0.0001
II-III	8 (5.8)	8 (47.1)	55 (100.0)	37 (0.0)	
Lymphatic invasion					
Negative	112 (81.2)	6 (35.3)	10 (18.2)	3 (8.1)	< 0.0001
Positive	26 (18.8)	11 (64.7)	45 (81.8)	34 (91.9)	
Venous invasion					
Negative	125 (90.6)	5 (29.4)	14 (25.4)	3 (8.1)	< 0.0001
Positive	13 (9.4)	12 (70.6)	41 (74.6)	34 (91.9)	

pT1: invasion of lamina propria or submucosa; pT2: invasion of muscularis propria; pT3: invasion of subserosa; pT4: penetration of serosa or invasion of adjacent structures.

**Figure 1:**
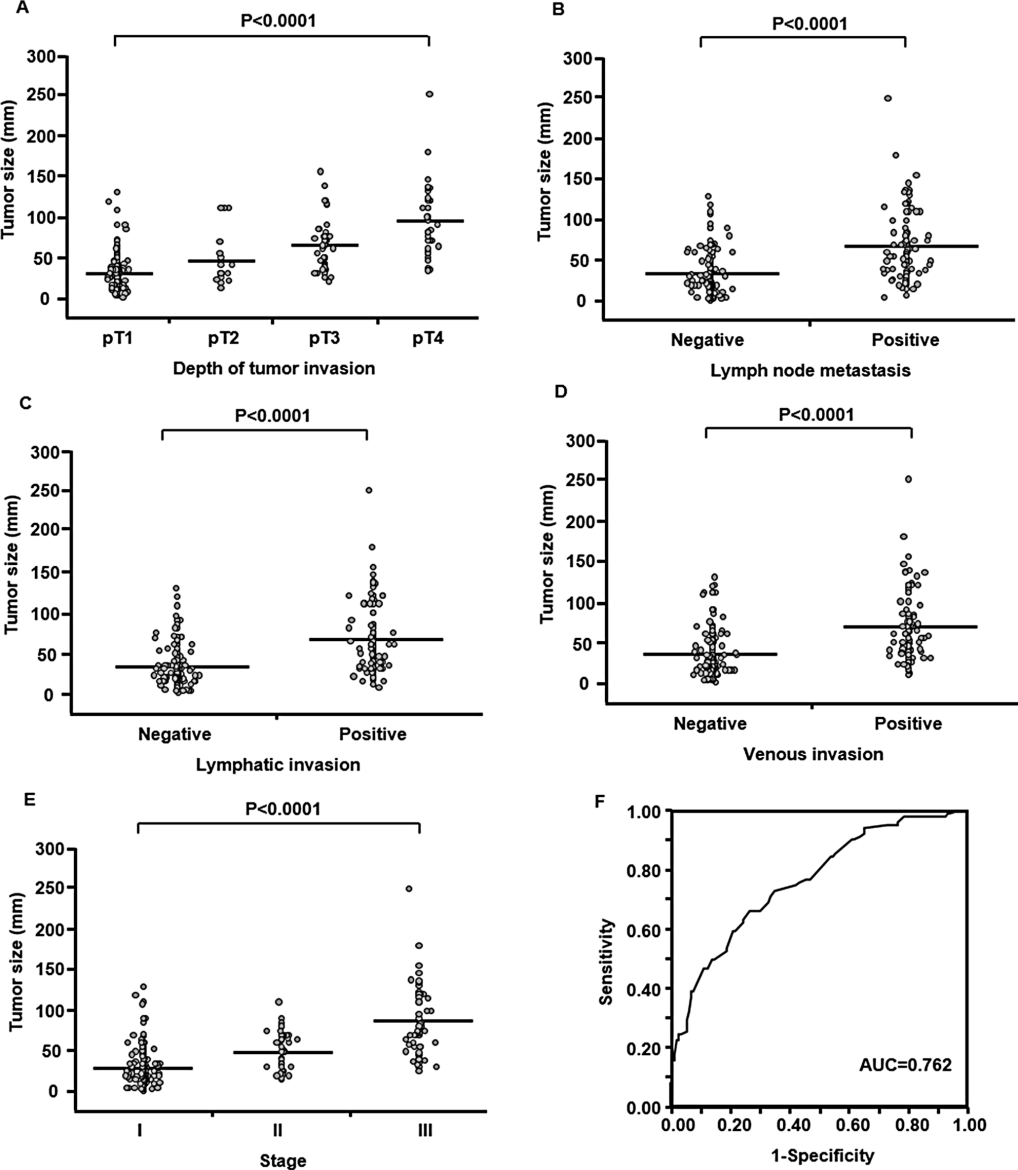
Relationship between tumor size and clinicopathological factors in 247 patients with resectable gastric cancer Tumor size correlated significantly with depth of tumor invasion (**A**), lymph node metastasis (**B**), lymphatic invasion (**C**), venous invasion (**D**) and stage (**E**). Horizontal bars indicate mean tumor size. The receiver operating characteristic curve for discriminating patients with nodal metastasis from patients without nodal metastasis based on tumor size (**F**).

Tumor size ranged between 1 and 250 mm in all patients with gastric cancer. Mean tumor size ± standard deviation (SD) was 49.4 ± 37.3 mm. Mean tumor size (± SD) in 138, 17, 55, and 37 patients with pT1, pT2, pT3, and pT4 tumors were 31.3 ± 22.7, 47.1 ± 33.1, 64.6 ± 30.1, and 95.4 ± 43.9 mm, respectively (Figure 1A). Tumor size correlated significantly with depth of tumor invasion (*P* < 0.0001). Mean tumor size (± SD) was 35.3 ± 25.1 mm in 143 patients with lymph node status N0 and 68.8 ± 42.4 mm in 104 patients with **≥** N1 (Figure 1B). Tumor size was significantly greater in patients with lymph node metastasis than in those without lymph node metastasis (*P* < 0.0001). In addition, tumor size correlated with presence or absence of lymphatic and venous invasions (*P* < 0.0001 each) (Figure 1C and 1D). Mean tumor size (± SD) was 31.5 ± 23.2 mm in 139 patients with stage I, 51.5 ± 23.0 mm in 44 patients with stage II, and 86.8 ± 41.9 mm in 64 patients with stage III (Figure 1E). Tumor size differed significantly between categorical stages (*P* < 0.0001).

Next, we investigated the potential utility of tumor size for predicting the presence or absence of lymph node metastasis using receiver operating characteristic (ROC) analysis. The area under the curve (AUC) value for discriminating patients with nodal metastasis from those without nodal metastasis based on tumor size was 0.762, setting a cut-off for tumor size of 45 mm (Figure 1F). The sensitivity and specificity of tumor size for predicting lymph node metastasis were 0.664 and 0.734, respectively. We therefore classified all patients into two groups according to tumor size **≥** 45 mm (*n* = 107); and < 45 mm (*n* = 140). This binary classification for tumor size was used in the following analyses.

### PTS grading system

A grading system of PTS was defined by both tumor depth and size. According to this grading system, all patients were categorized into three groups based on the depth status of tumor invasion and the cut-off value for tumor size, as follows: PTS 2, both deeper tumor invasion (pT2–T4) and greater tumor size **≥** 45 mm); PTS 1, either deeper tumor invasion or greater tumor size; and PTS 0, neither deeper tumor invasion nor greater tumor size.

According to this PTS grading system, 109 patients (44.1%) showed PTS 0, 60 patients (24.3%) showed PTS 1, and 78 patients (31.6%) showed PTS 2.

### PTS as a predictor of tumor progression

PTS correlated significantly with lymph node metastasis, lymphatic invasion, vascular invasion, and stage (*P* < 0.0001 each; Table 2).

**Table 2:** Relationship between primary tumor score and clinicopathological factors

Factor	Primary tumor score			*P*-value
	0 (*n =* 109)	1 (*n =* 60)	2 (*n =* 78)	
Sex				
Male	74 (67.9)	38 (63.3)	52 (66.7)	0.8337
Female	35 (32.1)	22 (36.7)	26 (33.3)	
Age (y)				
≤ 70	60 (55.1)	31 (51.7)	46 (59.0)	0.6883
>70	49(44.9)	29 (48.3)	32 (41.0)	
Depth of tumor invasion				
pT1	109 (100.0)	29 (48.4)	0 (0.0)	< 0.0001
pT2	0 (0.0)	11 (18.3)	6 (7.7)	
pT3	0 (0.0)	17 (28.3)	38 (48.7)	
pT4	0 (0.0)	3 (5.0)	34 (43.6)	
Lymph node metastasis				
Negative	92 (84.4)	35 (58.3)	16 (20.5)	< 0.0001
Positive	17 (15.6)	25 (41.7)	62 (79.5)	
Stage				
I	105 (96.3)	31 (51.7)	3 (3.8)	< 0.0001
II-III	4 (3.7)	29 (48.3)	75 (96.2)	
Lymphatic invasion				
Negative	91 (83.5)	29 (48.3)	11 (14.1)	< 0.0001
Positive	18 (16.5)	31 (51.7)	67 (85.9)	
Venous invasion				
Negative	98 (89.9)	34 (56.7)	15 (19.2)	< 0.0001
Positive	11 (10.1)	26 (43.3)	63 (80.8)	

pT1: invasion of lamina propria or submucosa; pT2: invasion of muscularis propria; pT3: invasion of subserosa; pT4: penetration of serosa or invasion of adjacent structures.

### PTS as a predictor of prognosis

**Figure 2:**
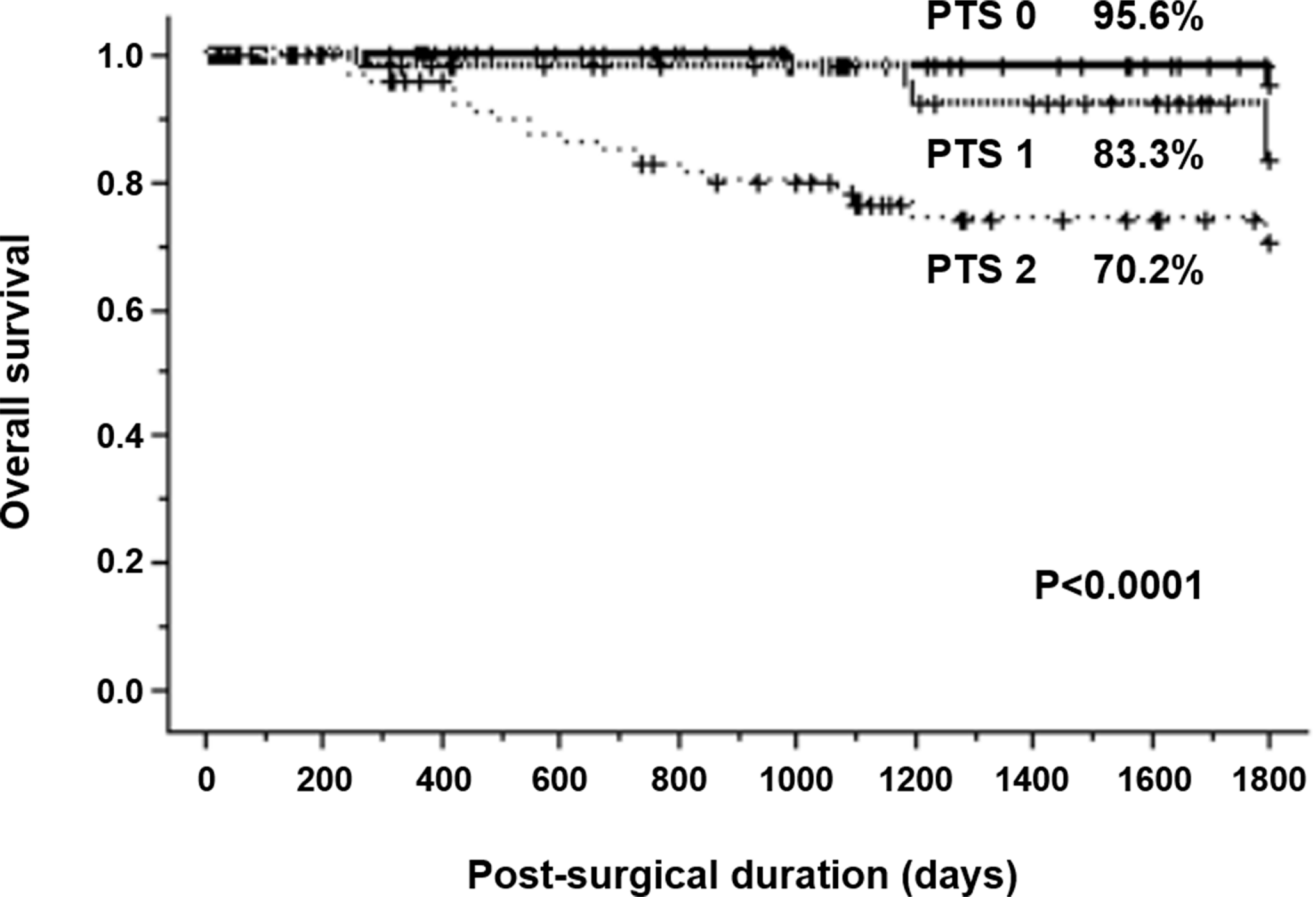
Kaplan–Meier survival curves for patients with resectable gastric cancer based on primary tumor score

Five-year survival rates were 95.6% for PTS 0, 83.3% for PTS 1, and 70.2% for PTS 2 (Figure 2). Significant differences in survival were seen according to PTS status (*P* < 0.0001).

### Uni- and multivariate analyses for survival

Tumor size, depth of tumor invasion, lymph node metastasis, stage, PTS, and serum concentrations of CEA and CA19-9 all correlated significantly with prognosis in univariate analysis (*P* = 0.0032, *P* < 0.0001, *P* = 0.0003, *P* < 0.0001, *P* < 0.0001, *P* = 0.0338, and *P* = 0.0043, respectively) (Table 3). Multivariate analysis demonstrated PTS alone as an independent prognostic factor (*P* = 0.0363; Table 4).

**Table 3:** Univariate analysis for survival

Independent factor	Hazard ratio	95% CI	P-value
Age (y)			
≤ 70/ >70	1.65	0.73–3.72	0.2217
Tumor size			
< 45 mm/ ≥ 45 mm	3.48	1.50–9.01	0.0032
Depth of tumor invasion			
pT1/pT2-T4	12.88	3.80–80.40	< 0.0001
Lymph node metastasis			
Negative/Positive	4.96	1.99–14.95	0.0003
Stage			
I/II-III	8.35	2.88–35.36	< 0.0001
Primary tumor score			
0-1/2	5.50	2.37–14.22	< 0.0001
Serum CEA levels (< 5 ng/mL)			
Negative/Positive	2.59	1.08–5.92	0.0338
Serum CA 19-9 levels (< 37 U/mL)			
Negative/Positive	3.96	1.59–9.12	0.0043

CI: confidence interval.

**Table 4:** Multivariate analysis for survival

Independent factor	Hazard ratio	95% CI	*P*-value
Lymph node metastasis			
Negative/Positive	2.28	0.78–7.72	0.1345
Primary tumor score			
0–1/2	2.86	1.07–8.53	0.0363
Serum CEA levels (< 5 ng/mL)			
Negative/Positive	1.95	0.74–4.90	0.1701
Serum CA 19-9 levels (< 37 U/mL)			
Negative/Positive	1.43	0.49–3.85	0.4963

CI: confidence interval.

## DISCUSSION

Initially, we assessed the clinical utility of tumor depth and size for predicting tumor progression in patients with resectable gastric cancer. We also proposed PTS as a new prognostic indicator determined solely from tumor depth and size. Finally, we evaluated the clinical significance of PTS as a predictor of tumor progression and prognosis in patients with stage I-III gastric cancer.

Depth of tumor invasion is a well-known prognostic factor in patients with gastric cancer. Unsurprisingly, this study demonstrated close relationships between tumor depth and several clinicopathological factors, such as lymph node metastasis and stage. Moreover, deeper tumor invasion was significantly associated with poor prognosis in the present study (data not shown). These results indicate the prognostic impact of tumor depth derived from primary tumor information in patients with gastric cancer.

We focused on tumor size as an additional prognostic factor in this study. Many investigators have demonstrated that greater tumor size correlates closely with increasing number of positive lymph nodes [6–8, 10, 12, 13, 15]. In the present study, tumor size correlated significantly with lymph node status. Interestingly, tumor size showed high sensitivity and specificity for discriminating between the presence and absence of lymph node metastasis. These findings suggest tumor size as a promising marker for predicting nodal status. In addition, it is important to estimate lymph node status by tumor size derived from primary tumor information alone for the clinical management of patients with resectable gastric cancer. Furthermore, a study of 2405 patients with gastric cancer by Zhao et al. reported that tumor size classified into five groups provided an independent prognostic factor according to multivariate analysis [15]. In that study, the 5-year survival rate was significantly lower in patients with tumor size **≥** 45 mm than in those with tumor size < 45 mm (*P* = 0.003; data not shown) [15]. Those results indicate that assessment of tumor size may be useful for clinical prediction of tumor aggressiveness, including prognosis.

The most noteworthy point of the present study was that we defined PTS calculated by tumor invasion and size derived from primary tumor information alone. At least metastatic status of lymph nodes and distant organs is needed to determine TNM stage. Ohashi et al. also reported the clinical potential of tumor index (TI) as a marker combining tumor depth and size in patients with gastric cancer [16]. They defined TI as pT category (pT1-4) multiplied by tumor size in millimeters [16]. That study identified a close association between TI and prominent clinicopathological prognostic factors, such as lymphovascular invasion, lymph node metastasis, and disease recurrence [16]. Moreover, they set a cut-off TI of 180 to determine the prognostic impact in two groups of patients [16]. In contrast, we categorized patients into three groups using our PTS grading system. The greatest advantage of the PTS grading system is the ability to strictly stratify patients into three categories of low, intermediate, and high malignant potential. Higher PTS correlated significantly with malignant tumor behaviors, such as the presence of lymph node metastasis and advanced stage. These findings suggest that the PTS grading system has potential as a tool for indicating pathological tumor progression.

Furthermore, the present study assessed the prognostic impact of the PTS grading system. Log-rank testing indicated that prognosis differed significantly among the three groups of PTS 0, 1 and 2 (*P* < 0.0001). Tumor size, depth of tumor invasion, and TNM stage were excluded from multivariate analysis due to confounding factors which might cancel out the efficacy of PTS. Then, only PTS was identified as an independent prognostic factor in multivariate analysis (*P* = 0.0363). Similarly, according to the study for TI, patients with TI **≥** 180 displayed significantly poorer prognosis than those with TI < 180 (*P* < 0.0001) [16]. These PTS and TI studies suggest that a score combining tumor depth and size determined from the primary tumor alone is useful to predict disease recurrence and prognosis in patients with gastric cancer. If PTS has potential for discriminating subclinical patients with high risk of disease recurrence, this grading system may assist in selecting patients for adjuvant chemotherapy in the postoperative management of gastric cancer.

The present study had several limitations. This preliminary study was based on a retrospective analysis of a small population (*n* = 247) in a single institution. Moreover, the median duration of follow-up was only 38 months. These limitations may have resulted in bias, which might have impacted several results in the study. Accordingly, larger validation studies with longer follow-up periods are required to strengthen our findings. We are presently planning further exploration of the PTS grading system in patients with other gastrointestinal tract cancers.

In conclusion, we proposed a promising prognostic score calculated from tumor depth and size, and demonstrated that PTS correlated closely with both tumor progression and prognosis in patients with gastric cancer. Stratification based on a PTS grading system might contribute to clinical planning of therapeutic strategies to optimize prognosis in patients with gastric cancer.

## MATERIALS AND METHODS

### Patients

Between December 1999 and January 2012, a total of 362 consecutive patients with gastric cancer underwent gastrectomy with lymphadenectomy at Kagoshima University Hospital (Kagoshima, Japan). Inclusion criteria for this study were as follows: 1) gastric adenocarcinoma confirmed by histopathology; 2) patients without endoscopic treatment; 3) patients without palliative resection; 4) patients without preoperative chemo- or radiotherapy; 5) patients without multiple gastric lesions; 6) patients without distant metastasis; and 7) patients without synchronous or metachronous cancer in other organs. After applying these criteria, 247 patients (164 men, 83 women; age range, 35–89 years; mean, 67 years) were enrolled in the present study. In surgical procedures, 150 (60.7%), 24 (9.7%), and 73 (29.6%) patients underwent distal gastrectomy, proximal gastrectomy, and total gastrectomy, respectively. Moreover, the mean number of dissected lymph nodes was 31.5. Patients were classified and staged based on the TNM classification of gastric carcinoma established by the UICC [5]. Table 5 shows the clinicopathological background of participating patients. All patients were followed-up every 3-6 months after surgery by regular clinical examinations at Kagoshima University Hospital, including tumor marker studies (CEA and CA19-9), radiography, ultrasonography, and computed tomography. The median duration of follow-up was 38 months (range, 1–123 months).

**Table 5:** Clinicopathological factors in 247 patients with resectable gastric cancer

Sex	
Male	164
Female	83
Age (y)	
≤ 70	137
> 70	110
Tumor location	
Upper	62
Middle	103
Lower	82
Depth of tumor invasion	
pT1	138
pT2	17
pT3	55
pT4	37
Lymph node metastasis	
Negative	143
Positive	104
Stage	
I	139
II	44
III	64
Lymphatic invasion	
Negative	131
Positive	116
Venous invasion	
Negative	147
Positive	100
Histological type	
Differentiated	104
Undifferentiated	143
Serum CEA levels (< 5 ng/mL)	
Negative	196
Positive	49
Unknown	2
Serum CA 19-9 levels (< 37 U/mL)	
Negative	211
Positive	31
Unknown	5

pT1: invasion of lamina propria or submucosa; pT2: invasion of muscularis propria; pT3: invasion of subserosa; pT4: penetration of serosa or invasion of adjacent structures.

This retrospective observational study was approved by the Ethics Committee of Kagoshima University and all patients provided written informed consent to the use of their information.

### Assessment of tumor depth and size

Depth of tumor invasion was assessed based on the TNM classification of gastric carcinoma [5]. Accordingly, tumors were pathologically divided into four T-categories: pT1, pT2, pT3, and pT4.

Tumor size was measured according to the Japanese Classification of Gastric Carcinoma (3rd English edition) [17]. The resected specimen was opened along the greatest curvature and placed on a flat board. The maximum tumor diameter was recorded as tumor size and used for further analysis in this study. Furthermore, tumor spread was pathologically reconfirmed.

### Statistical analysis

The relationship between tumor depth and categorical clinicopathological factors was assessed using the Chi-squared test or Fisher’s exact test. Differences in the relationship between tumor size and clinicopathological factors were evaluated using the Wilcoxon rank-sum test. ROC curves were constructed and the AUC was calculated to assess the predictive power of tumor size to discriminate patients with lymph node metastasis from those without lymph node metastasis. The relationship between PTS and clinicopathological features was analyzed using the Chi-squared test or Fisher’s exact test. Kaplan–Meier survival curves were generated and prognostic differences were determined using log-rank testing. Prognostic factors were assessed by uni- and multivariate analyses (Cox proportional hazards regression modeling). All data were analyzed using SAS statistical software (SAS Institute, Cary, NC). A value of *P* < 0.05 was considered statistically significant.
